# Microbial Involvement in Carbon Transformation via CH_4_ and CO_2_ in Saline Sedimentary Pool

**DOI:** 10.3390/biology10080792

**Published:** 2021-08-17

**Authors:** Weronika Goraj, Anna Szafranek-Nakonieczna, Jarosław Grządziel, Cezary Polakowski, Mirosław Słowakiewicz, Yanhong Zheng, Anna Gałązka, Zofia Stępniewska, Anna Pytlak

**Affiliations:** 1 Institute of Biological Sciences, The John Paul II Catholic University of Lublin, Konstantynów 1 I, 20-708 Lublin, Poland; anna.szafranek-nakonieczna@kul.pl; 2 Department of Agricultural Microbiology, Institute of Soil Science and Plant Cultivation—State Research Institute (IUNG-PIB), Czartoryskich 8, 24-100 Puławy, Poland; jgrzadziel@iung.pulawy.pl (J.G.); agalazka@iung.pulawy.pl (A.G.); 3 Institute of Agrophysics, Polish Academy of Sciences, Doświadczalna 4, 20-290 Lublin, Poland; c.polakowski@ipan.lublin.pl (C.P.); apytlak@ipan.lublin.pl (A.P.); 4 Faculty of Geology, University of Warsaw, Żwirki i Wigury 93, 02-089 Warszawa, Poland; m.slowakiewicz@uw.edu.pl; 5 Institute of Geology and Petroleum Technologies, Kazan Federal University, Kremlovskaya 18, 420008 Kazan, Russia; 6 State Key Laboratory of Continental Dynamics, Department of Geology, Northwest University, Xi’an 710069, China; zhengnwu@163.com; 7 Department of Biochemistry and Environmental Chemistry, The John Paul II Catholic University of Lublin, Konstantynów 1 I, 20-708 Lublin, Poland; stepz@kul.pl

**Keywords:** methane, methane oxidation, methanogenesis, aerobic respiration, anaerobic respiration, saline environments, NGS, microbial community, *Bacillus*

## Abstract

**Simple Summary:**

Methane and carbon dioxide are commonly found in the environment and are considered the most important greenhouse gases. Transformation of these gases is in large carried by microorganisms, which occur even in extreme environments. This study presents methane-related biological processes in saline sediments of the Miocene Wieliczka Formation, Poland. Biological activity (carbon dioxide and methane production or methane oxidation), confirmed by stable isotope indices, occurred in all of the studied Wieliczka rocks. CH_4_-utilizing microbes constituted 0.7–3.6% while methanogens (represented by *Methanobacterium)* only 0.01–0.5% of taxa present in the Wieliczka Salt Mine rocks. Water activity was the key factor regulating microbial activity in saline subsurface sediments. Generally, CO_2_ respiration was higher in anaerobic conditions while methanogenic and methanotrophic activities were dependent on the type of rock.

**Abstract:**

Methane and carbon dioxide are one of the most important greenhouse gases and significant components of the carbon cycle. Biogeochemical methane transformation may occur even in the extreme conditions of deep subsurface ecosystems. This study presents methane-related biological processes in saline sediments of the Miocene Wieliczka Formation, Poland. Rock samples (W2, W3, and W4) differed in lithology (clayey salt with veins of fibrous salt and lenses of gypsum and anhydrite; siltstone and sandstone; siltstone with veins of fibrous salt and lenses of anhydrite) and the accompanying salt type (spiza salts or green salt). Microbial communities present in the Miocene strata were studied using activity measurements and high throughput sequencing. Biological activity (i.e., carbon dioxide and methane production or methane oxidation) occurred in all of the studied clayey salt and siltstone samples but mainly under water-saturated conditions. Microcosm studies performed at elevated moisture created more convenient conditions for the activity of both methanogenic and methanotrophic microorganisms than the intact sediments. This points to the fact that water activity is an important factor regulating microbial activity in saline subsurface sediments. Generally, respiration was higher in anaerobic conditions and ranged from 36 ± 2 (W2_200%t.w.c_) to 48 ± 4 (W3_200%t.w.c_) nmol CO_2_ gdw^−1^ day^−1^. Methanogenic activity was the highest in siltstone and sandstone (W3, 0.025 ± 0.018 nmol CH_4_ gdw^−1^ day^−1^), while aerobic methanotrophic activity was the highest in siltstone with salt and anhydrite (W4, 220 ± 66 nmol CH_4_ gdw^−1^ day^−1^). The relative abundance of CH_4_-utilizing microorganisms (*Methylomicrobium*, *Methylomonas*, *Methylocystis*) constituted 0.7–3.6% of all taxa. Methanogens were represented by *Methanobacterium* (0.01–0.5%). The methane-related microbes were accompanied by a significant number of unclassified microorganisms (3–64%) and those of the *Bacillus* genus (4.5–91%). The stable isotope composition of the CO_2_ and CH_4_ trapped in the sediments suggests that methane oxidation could have influenced δ^13^C_CH4_, especially in W3 and W4.

## 1. Introduction

Methane (CH_4_) and carbon dioxide (CO_2_) are important contributors to the global carbon cycle and major greenhouse gases (GHG) in the atmosphere [1]. In 2019, the global average atmospheric mixing ratio of CO_2_ was 409.8 ppm [2] while of CH_4_ was 1.875 ppb [3]. Methane is a particular cause for concern because the global warming potential (GWP) for CH_4_ (depending on whether various indirect climate effects are included) is estimated at 28–36 times that of CO_2_ [4,5], and thus despite the relatively low concentration, methane is responsible for binding about 17% of the thermal energy in the atmosphere [6,7]. Considering the trends in global GHG atmospheric mixing ratios, each uncertainty concerning their sources and sinks should be tackled.

As suggested by recent studies [8,9,10,11], the deep biosphere comprise a reservoir of microorganisms that may have significant implications for biogeochemical carbon cycling. There is growing evidence that biological GHG transformation also occurs in the harsh conditions of the deep subsurface [12,13,14,15]. Despite this, still little is known about the role of subterranean saline ecosystems, which are widespread throughout the world [16]. The available literature is scarce [17,18] and therefore, microbial activities that take part in methane transformation and occur in saline geological formations are of great interest.

It has recently been shown that the Wieliczka saline sediments have a potential to buffer some of geological methane emissions through aerobic methanotrophic activity [19]. It should be noted, however, that subsurface environments also harbour dormant microorganisms which, under favourable conditions, become active [13,20]. With these in mind, we have analysed the response of the microbial communities present in the Wieliczka Formation sediments to various moisture and aeration conditions. The microbiological studies included determination of biological activity, i.e., methane formation, methane utilization and respiration (in aerobic and anaerobic conditions) as well as the identification of microbial inhabitants. Furthermore, δ^13^C isotopic ratios were determined to evaluate the nature (biogenic/abiogenic) of methane trapped in the rock pores. Understanding the biodiversity and the processes taking place in saline sediments can better decipher microbial life of the deep biosphere, which is still not well understood.

## 2. Materials and Methods

### 2.1. Study Site

**Figure 1:**
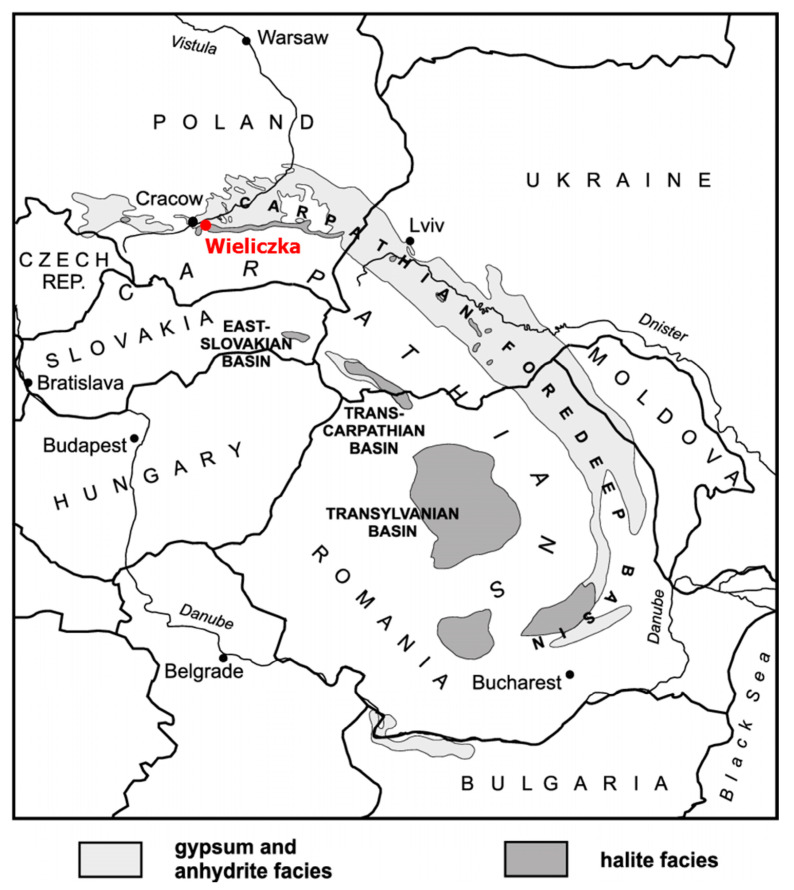
Location of Miocene evaporite deposits in the Carpathian Foredeep (adapted from Babel [27]).

Wieliczka Formation (49°59′ N 020°03′ E) is located in southern Poland, ca. 13 km northeast of Kraków and is accessible via the Wieliczka Salt Mine (Figure 1). It extends over a length of ca. 10 km and covers an area of the Carpathian Foredeep and adjacent Carpathian Foreland [21]. The formation is built of Badenian (16.3–12.8 Ma, [22] rock salt with intercalations of claystone and minor sulphates dated at 13.81 ± 0.08 Ma [23]. Its origin is linked with the salinity crisis that, according to various estimates, lasted for between 20 ka [24] and 600 ka [23] and led to the disappearance of large marine basins. Three samples (W2, W3, W4) of sedimentary rocks surrounding the salt deposit in the Wieliczka Salt Mine were collected at a depth of 110–130 m. Rock samples differed in lithology and the accompanying salt type (Table 1) [19,25,26].

### 2.2. Sample Collection and Processing

Samples used for microbiological and biogeochemical assays were collected as solid clumps (ca. 4 kg), which were extracted from freshly excavated surfaces of the Wieliczka Formation sediments, were immediately put into sterile plastic containers and tightly sealed. Simultaneously, triplicate fragmented sub-samples (450–500 g) were placed in hermetic glass jars with a septum to enable analysis of the isotopic composition of CO_2_ and CH_4_ released from the rock pores. Desorption jars were flushed with N_2_ and samples were analysed after 10 days of desorption. Prior to chemical and physical analyses, rock samples were crushed to a diameter < 2 mm. The clumps used for determining methane production and utilization potentials or DNA isolation were externally sterilized by UV irradiation and flaming as previously described [28].

### 2.3. Isotopic Analysis

Stable carbon isotopic analyses of CO_2_ and CH_4_ were carried out using an isotope ratio mass spectrometer (IRMS) ThermoElectronics DELTA V Advantage with a continuous gas flow connected to an Automated Trace Gas Pre-Concentrator (Thermo Electronics) as described previously [29]. δ^13^C values are reported as ‰ on the Peedee belemnite (PDB) scale. Reproducibility within runs was 0.05‰.

### 2.4. Present-Day Methane and Carbon Dioxide Turnover

Microbial methane turnover (methanogenesis and methanotrophy) as well as carbon dioxide production (respiration) were determined by 16- and 56-day (aerobic and anaerobic respiration) and 77- and 900-day (methanotrophic and methanogenic activity) incubations of crushed rock (15 g) in sterile dark glass bottles (60 cm^3^). Two moisture variants were applied, i.e., natural (W2_n_-4_n_) and 200% of total water capacity (t.w.c) (achieved by the addition of sterile degassed water). The 200% t.w.c. was aimed to be a proxy of seabed conditions. The bottles were hermetically closed with rubber septa and capped with an aluminium cap. Incubations aimed at determining anaerobic activity were processed in an anaerobic glovebox chamber with a nitrogen gas atmosphere (Labconco, Kansas City, MO, USA). For the methanotrophic incubation, an initial concentration of ca. 10% (*v*/*v*) CH_4_ was obtained by replacing an appropriate volume of air or nitrogen (respectively for aerobic and anaerobic incubation) with methane. A detailed outline of the applied treatments is summarized in Table 2. For each treatment, three independent replicates were incubated. Simultaneously, control samples, three times autoclaved (for 30 min at 121 °C and 15 psi) rock, were prepared and incubated in the same conditions.

Changes in methane and carbon dioxide concentrations over time were determined using a Varian CP-3800 gas chromatograph equipped with flame ionization (FID, 200 °C) and thermal conductivity (TCD, 120 °C) detectors [13,30,31]. CH_4_ production and CO_2_ release rate (respiration) were determined on the basis of increase in methane or carbon dioxide concentration over time while methane oxidation was based on methane decrease and expressed respectively in nmol CH_4_ gdw^−1^ day^−1^ and nmol CO_2_ gdw^−1^ day^−1^.

### 2.5. Microbial Diversity and Molecular Techniques

Rock samples (10 g), aseptically collected from the interior of the clumps, were transferred directly into the bead-beating solution of the DNeasy PowerMax Soil Kit (Qiagen, MD, USA) and processed according to the manufacturer’s protocol. The V3-V4 hypervariable regions of the bacterial 16S rRNA gene were amplified using the primer pair: 341F-CCTACGGGNGGCWGCAG and 785R-GACTACHVGGGTATCTAATCC. The targeted gene regions have been shown to be the most suitable for Illumina sequencing [32]. Each sample was amplified with NEBNext^®^ High-Fidelity 2xPCR Master Mix (New England BioLabs) according to the manufacturer’s instructions. Paired-end (2 × 250 nt) sequencing was performed with an Illumina MiSeq by Genomed S.A. (Warsaw, Poland) and following the manufacturer’s run protocols (Illumina, Inc., San Diego, CA, USA).

### 2.6. Data Analysis

Demultiplexed fastq files were processed using the DADA2 (1.12) package [33] in R software (3.6.0) [34]. Forward and reverse reads were trimmed to 250 bp, primer sequences were removed from all reads. Filtering parameters were as follows: maxN = 0, maxEE for both reads = 3, truncQ = 2. MaxEE corresponds to the maximum expected errors. Expected errors are calculated from the quality score (EE = sum (10^(−Q/10)). The error rates were estimated by learnErrors using one million reads. Sequences were dereplicated using derepFastq with default parameters, and exact amplicon sequence variants (ASV) were resolved using dada. Next removeBimeraDenovo was used to remove chimeric sequences. After the filtration steps, 93703–107115 (mean = 99208) of the reads were left for further analysis. Taxonomy was assigned against the latest version of the modified RDP database [35] using IDTAXA [36] on the sequences table resulting from the DADA2 workflow described above. The results were converted and imported into the phyloseq (1.22.3) package [37]. Sequences belonging to chloroplast or mitochondrial DNA were removed. Subsequently, for further analysis, the total number of reads for the individual taxa were converted to a percentage, assuming the sum of all taxa in the individual samples as 100%. On average, 69.79% of all reads, which were correctly classified to the genus level, were aggregated and their abundances were summed up. Unclassified reads were clustered using vsearch [38] implemented in seed software, version 2.1 [39] at a 97% similarity level. Each of 317 clustered groups of unclassified reads were then named from Unclassified_WGW0001 to Unclassified_WGW0317 and merged with the previous table (containing reads classified to the genus level). The unclassified sequences constituting >1% were analysed separately using BLASTN (NCBI).

Evolutionary history was inferred using the Neighbour-Joining method [40]. The bootstrap consensus tree inferred from 500 replicates is taken to represent the evolutionary history of the taxa analysed [41]. Branches corresponding to partitions reproduced in less than 50% of the bootstrap replicates were collapsed. The percentage of replicate trees in which the associated taxa clustered together in the bootstrap test (500 replicates) are shown next to the branches [41]. The evolutionary distances were computed using the number of differences method [42] and are in the units of number of base differences per sequence. The analysis involved 42 nucleotide sequences. All positions containing gaps and missing data were eliminated. There were a total of 256 positions in the final dataset. Evolutionary analyses were conducted in MEGA X [43].

This approach enabled the statistical processing of true alpha and beta diversity, regardless of whether a sequence exists in the reference database or not. In total, 443 unique taxa (at the genus level plus unclassified clusters) were detected in all samples. Alpha diversity indices were calculated using the phyloseq package.

NGS sequence data have been deposited in the NCBI Sequence Read Archive (SRA) database under BioProject’s ID: PRJNA596092.

## 3. Results

The studied samples represented various lithotypes (clayey salt, siltstone and sandstone, siltstone with veins of fibrous salt which refer to samples W2, W3 and W4, respectively). Most importantly, they were characterized by variable salinity. The highest NaCl content (calculated for pore water at in situ moisture) was found in W2 (221 g L^−1^) and the lowest salt concentration was observed in W4 (72 g L^−1^) and W3 (25 g L^−1^) (Table 3). Other factors determining the living conditions of microorganisms, i.e., availability of basic nutrients, were similar in all the analysed materials [19] (Table 3).

### 3.1. Methane Turnover and Respiration Rates

**Figure 2:**
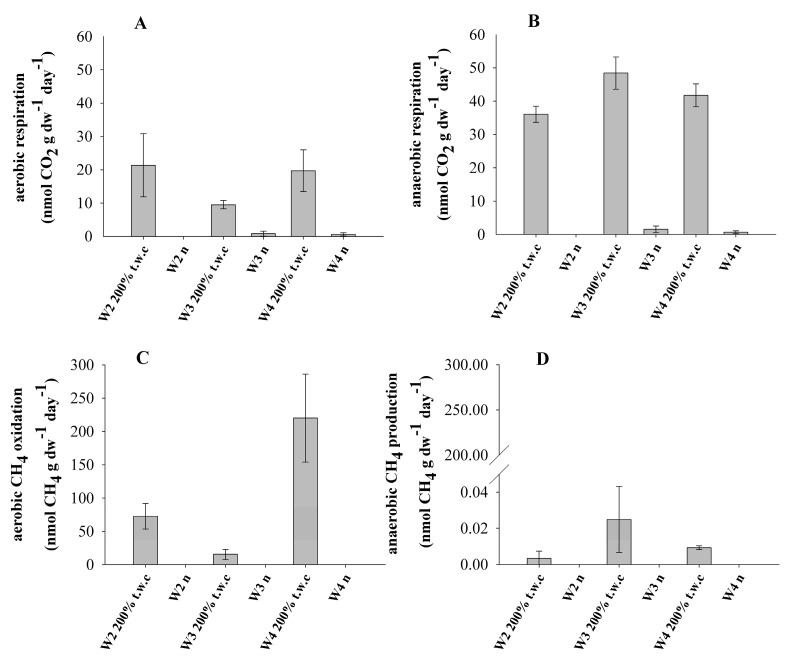
Microbial activity ((**A**)—aerobic respiration; (**B**)—anaerobic respiration; (**C**)—aerobic CH_4_ oxidation; (**D**)—anaerobic CH_4_ production) in marine sediments under different moisture (natural—n and saturated—200% t.w.c.) and aeration conditions at 20 °C (error bars represent standard deviation, n = 3).

The studied samples were subjected to long-term incubations aimed at determining the rate of methane turnover and respiration. Examples of gas dynamics are included in the supplementary materials (Figure S1). The treatments applied included variable moisture conditions and terminal electron availability (Table 3). It was found that both at aerobic and anaerobic conditions the main factor regulating microbial activity was moisture. Microbial activity of the pristine samples was very low (Figure 2) while CH_4_ and CO_2_ concentrations in the water-saturated samples were several times higher (Figure 2).

Methane production occurred in all of the studied samples but only in the water-saturated conditions. The methanogenic activity, calculated from the linear increase in methane concentration was highest in W3 (0.025 ± 0.018 nmol CH_4_ gdw^−1^ day^−1^), compared with much lower values in other samples (Figure 2D). Aerobic methanotrophic activity, was detected only in the water-saturated conditions and reached 220 ± 66 nmol CH_4_ gdw^−1^ day^−1^ in W4 (Figure 2C). Anaerobic methane oxidation was not detected in any of investigated moisture conditions. At the same time, no changes in CO_2_ and CH_4_ concentrations were recorded in the control samples.

Biological activity, expressed as carbon dioxide production, was observed in all samples except W2_n_. Generally, anaerobic respiration was higher than aerobic. Under water-saturated conditions, daily CO_2_ production ranged from 9.5 ± 1 (W2_200%t.w.c_) to 21 ± 9 nmol CO_2_ gdw^−1^ day^−1^ (W4_200%t.w.c_) in aerobic conditions and from 36 ± 2 (W2_200%t.w.c_) to 48 ± 4 (W3_200%t.w.c_) nmol CO_2_ gdw^−1^ day^−1^ in anaerobic conditions. At natural moisture, only W3_n_ and W4_n_ rock samples revealed detectable CO_2_ quantities, which correspond to a daily respiration of between 0.59 ± 0.5 and 0.82 ± 0.7 nmol CO_2_ gdw^−1^ day^−1^ in aerobic conditions and from 0.65 ± 0.4 to 1.5 ± 1 nmol CO_2_ gdw^−1^ day^−1^ in anaerobic conditions (Figure 2A,B).

### 3.2. Microbial Identification

#### 3.2.1. Biodiversity

The diversity and richness indices of all samples were calculated in an effort to illustrate the complexity of the microbial communities found in the saline sediments (Table 4).

The smallest number of ASV were identified in the W2 sample (77); however, the taxa were evenly distributed and as a result, the values of the Shannon index were as high 3.234. In the W3 sample, there were more ASV identified (199) but most of them were represented by a low number of reads. *Bacillus* was a predominant component of the W3 microbial community and, therefore, the Shannon index calculated for this sample was very low (0.621). The sample characterized by the highest biodiversity was W4, where 242 unique ASV were found. The Shannon index for W2 was 4.987. Simpson (inverse Simpson) index values showed a similar tendency as in the case of the Shannon index (Table 4).

#### 3.2.2. Microbial Community Structure

**Figure 3:**
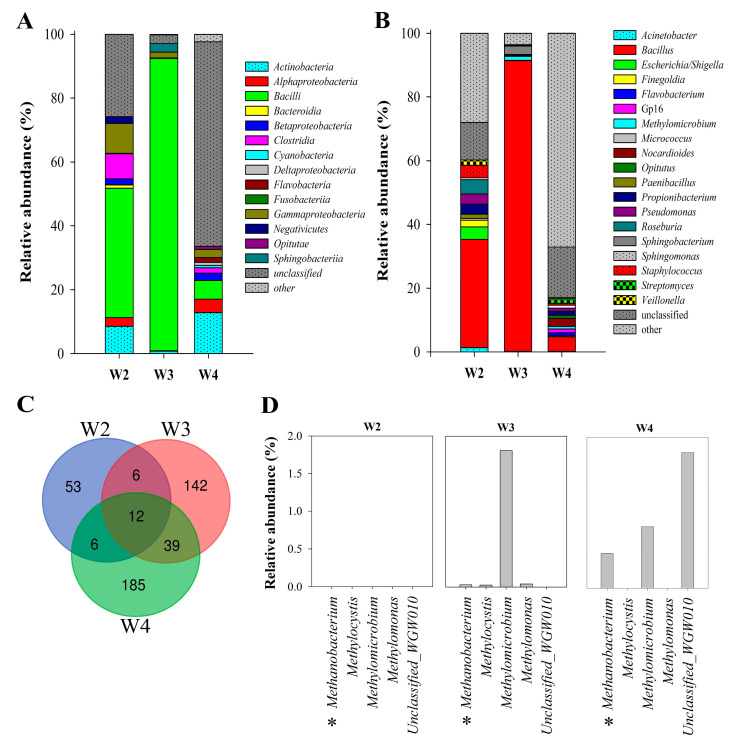
Microbial community structure of Miocene sulphate rocks by 16S rRNA gene amplicon analysis based on Next Generation Sequencing at the class and genus level. Relative abundance (%) of the top 10 classes (**A**) and genera (**B**) of microbes. Venn diagram of overlapping all bacterial communities at the genus level (**C**). Participation of methanotrophic and methanogenic microorganisms (*) in the microbial community (**D**).

In general, the investigated samples were dominated by *Bacteria* (26–97%) and unclassified microorganisms (3–64%). In the W3 and W4 samples, aside from the *Bacteria* domain, *Archaea* (to 0.5%) were also observed. Taxonomic analysis of NGS results indicated dominance by the phylum *Firmicutes* (50–92%) in the W2 and W3 samples, whereas W4 was dominated by unclassified microorganisms (64%). *Proteobacteria* and *Actinobacteria* were also found in large numbers (10–14% and 8–10% respectively) in W2 and W4. Analysis of deeper taxonomic levels revealed 14 different bacterial classes present in the Wieliczka Formation sediments (Figure 3A). Figure 3A presents the top 10 classes for each sample, while other classifications were grouped in the “other” category. The most abundant class in the W2 and W3 samples was *Bacilli* (40 and 91%), while in the W4 sample it made up only ca. 6%. Apart from the unclassified microorganisms (64%), the W4 community comprised a substantial proportion of *Actinobacteria* (13%). In W3, *Bacilli* were accompanied by *Sphingobacteriia* (3%) and *Gammaproteobacteria* (2%), whereas in W2, *Clostridia*, *Gammaproteobacteria* and *Actinobacteria* were at a similar level (8–9%).

Figure 3C shows all 443 ASV identified in the studied rock samples. At the genus level, the communities differed significantly and only 12 taxa were common to all samples. However, analysing the 10 most abundant taxa, it was found that there were only three common types (*Acinetobacter*, *Bacillus* and *Escherichia*/*Shigella*) (Figure 3B). The unique taxa in the W2 sample were *Finegoldia*, *Paenibacillus*, *Roseburia*. W4 included only one exceptional taxon—*Opitutus*. In general, the relative abundance of dominant genera corresponds with taxon distribution on the class level (Figure 3A,B). The genera belonging to Archaea were *Methanobacterium* (up to 0.5%) and *Halorientalis* (0.014%).

The relative abundance of CH_4_-utilizing microorganisms was 0.7–3.6%. In this group, both obligatory and facultative methanotrophs could be distinguished. Obligatory methanotrophic bacteria were represented by *Methylomicrobium* spp. (1.3% in W3 and 0.8% in W4) and *Methylomonas* (≤0.03%). Among the facultative methanotrophs, only *Methylocystis* was identified on the genus level although reads described as Unclassified_WGW010 can also be added to this group, as they showed 100% identity with uncultured representatives of *Methylocystaceae* (EF072118.1). *Methylocystis* was most abundant in the W4 sample (1.8%). Interestingly, in W3 and W4 samples, sequences affiliated to methanotrophic and methanogenic microorganisms co-occurred. The methanogens were represented by *Methanobacterium* which comprised 0.01 to 0.5 % of the microbial communities found in W3 and W4, respectively (Figure 3D).

**Figure 4:**
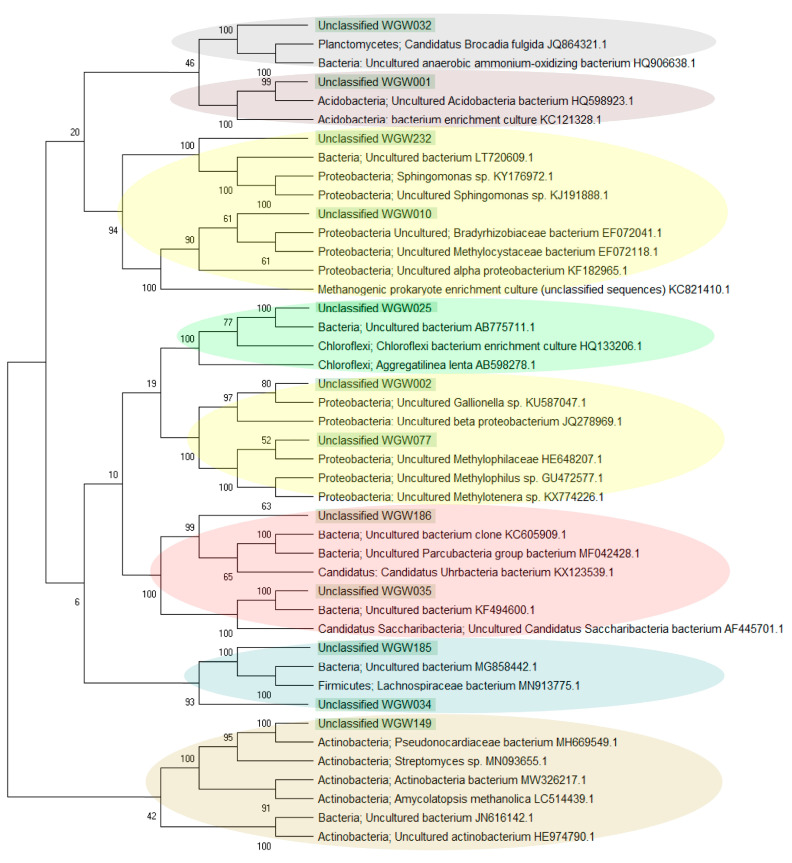
Evolutionary relationships of the unclassified sequences, where the numbers above the branches indicate the bootstrap value. Phylogenetic tree determined by the Neighbour-joining method using MEGA-X software. The clades belonging to the same taxa are marked with one colour.

**Figure 5:**
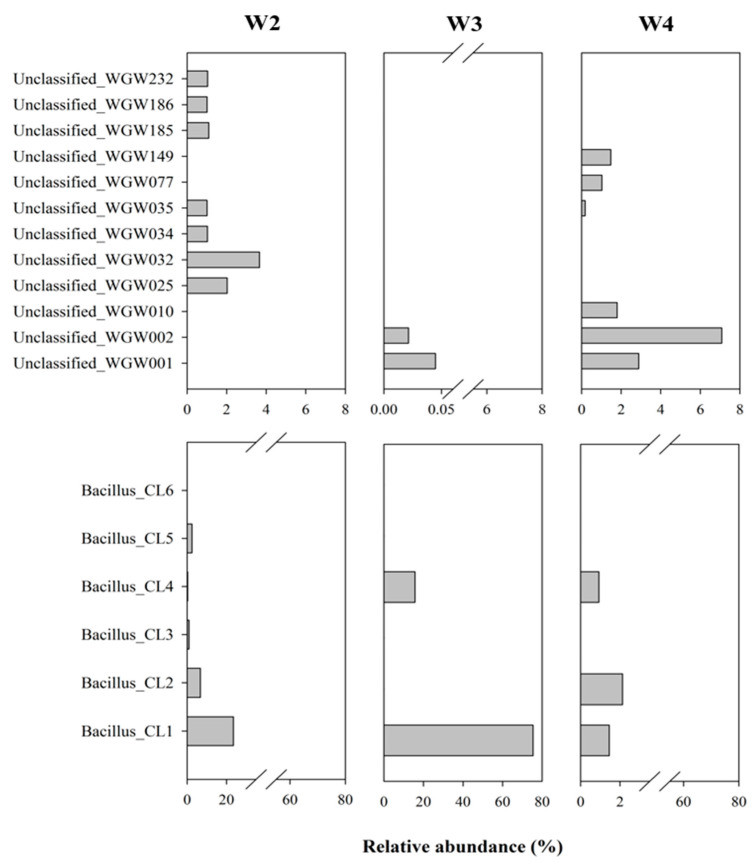
Relative abundance (%) of the unclassified sequences and sequences assigned to *Bacillus* in microbial community structure of marine sedimentary rocks.

Among the 443 ASV detected in the studied saline sediments, as many as 317 were assigned as unclassified. The sequences constituting > 1% were analysed separately using BLASTN (NCBI). Unclassified_WGW032, WGW001, WGW025 and WGW149 formed separate taxa and were not related to each other or to the other analysed unclassified sequences, they occurred mainly in samples W2 and W4 but also in W3 (Unclassified_WGW001). The remaining sequences were grouped into 4 clades. The sequence Unclassified_WGW232, which was identified in the W2 sample, was similar to Unclassified_WGW010 found in W4. Unclassified_WGW186 (found in W2) and Unclassified_WGW035 (found in W2 and W4) were grouped into the second clade. It was also shown that two other unclassified sequences (Unclassified_WGW002 and WGW077) present in W3 and W4 samples were similar to each other. In the last clade, the sequences Unclassified_WGW185 and WGW034 identified in W2 were grouped (Figure 4 and Figure 5). Evolutionary relationships with high bootstrap value indicate a potential affiliation of unclassified sequences to selected taxa at the phylum level, i.e., *Planctomycetes* (Unclassified_WGW032), *Acidobacteria* (Unclassified_WGW001), *Proteobacteria* (Unclassified_WGW232, WGW010, WGW002 and WGW077), *Chloroflexi* (Unclassified_WGW025), *Candidatus* (Unclassified_WGW186 and WGW035), *Firmicutes* (Unclassified_WGW185 and WGW034) and *Actinobacteria* (Unclassified_WGW149) (Figure 4).

**Figure 6:**
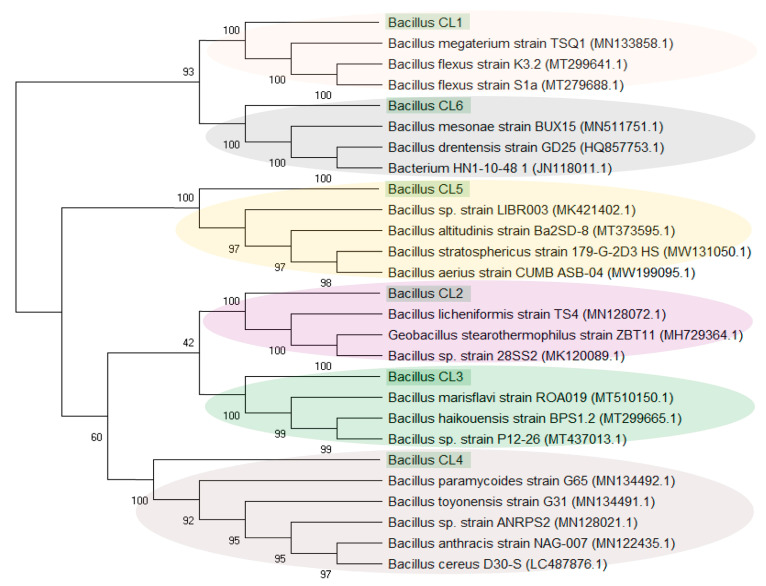
Evolutionary relationships of *Bacillus* clusters, where the numbers above the branches indicate the bootstrap value. Phylogenetic tree determined by the Neighbour-joining method using MEGA-X software. The clades are marked with colours.

Similarly, due to their high percentage (up to 91% in W3), the sequences assigned to *Bacillus* were analysed separately. It was found that the sequences classified as *Bacillus* (21 different, unique sequences) were grouped into six clusters (designated as *Bacillus*_CL1-6). Figure 5 and Figure 6 present the percentage of the occurrence of individual clusters in the studied samples (sequences > 1%) and the phylogenetic tree of these clusters. *Bacillus*_CL1, CL5 and CL6 clusters were grouped into one clade, wherein *Bacillus*_CL1 and CL6 were most closely related to each other. However, *Bacillus*_CL3 and CL4 formed a second clade and CL2 are less related than the rest of the *Bacillus* sequences. (Figure 6). This unique *Bacillus* CL1 clade accounted for more than 23% and 75% of all sequences assigned to *Bacillus* in samples W2 and W3, respectively. In the W2 sample, in addition to CL1, the sequences assigned to CL2-CL5 (0.34–6.7%) were also found. In the W3 sample, *Bacillus*_CL4 (16%), CL2 (0.09%) and CL6 (0.02 %) also occurred. The smallest diversity among *Bacillus* sequences was demonstrated in sample W4 where clades *Bacillus*_CL1 CL2 and CL4 (1–2%) were present (Figure 5).

### 3.3. Stable Methane and Carbon Dioxide Isotopes

**Figure 7:**
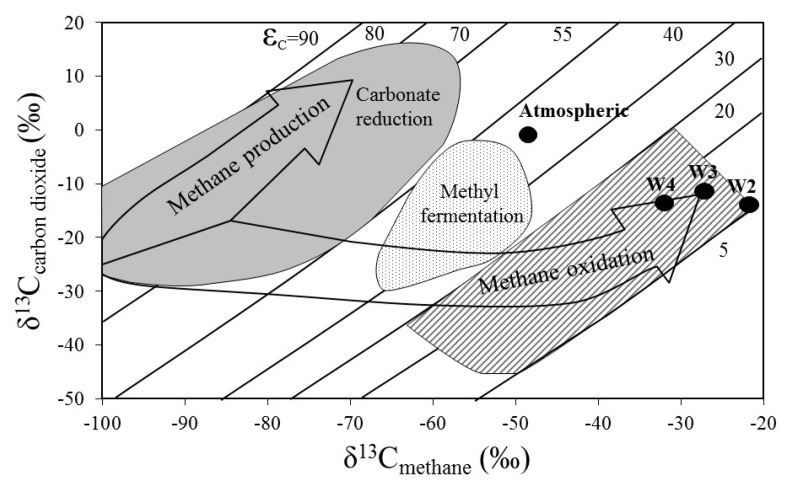
Methane oxidation and production pathway in the Wieliczka Formation sediments through stable δ^13^C_methane_ versus δ^13^C_carbon dioxide_ isotopes (adapted from Whiticar [45]).

The carbon isotope ratios of gases desorbed from rock samples exhibited a narrow range of the δ^13^C values of CH_4_ (−21 to −33‰) and CO_2_ (−12 to −14‰) (Figure 7). The isotopic indices are distinct from those characterizing atmospheric gases. The δ^13^C_CH4_ values suggest a thermogenic origin of methane [44] and transportation from deeper strata. Comparison of the obtained values with the genetic diagram proposed by Whiticar (1996) suggests that the process that influenced carbon fractionation was rather biological methane oxidation than methanogenesis [45]. The greatest δ^13^C fractionation was found in the W4 sample, while the smallest in W2.

## 4. Discussion

### 4.1. The Role of Salinity in Methane Metabolism

Microbial activity in the sediments of the Wieliczka Formation was highly stimulated by moisture (Figure 2). It is thus clear that the microbiota of the studied sediments are not well adapted to current (dry) subsurface conditions. One putative explanation is that it contains a vast array of dormant microorganisms, being a remnant of the sedimentation period when water content in sediments was high. Another explanation is later intrusion with meteoric waters.

**Figure 8:**
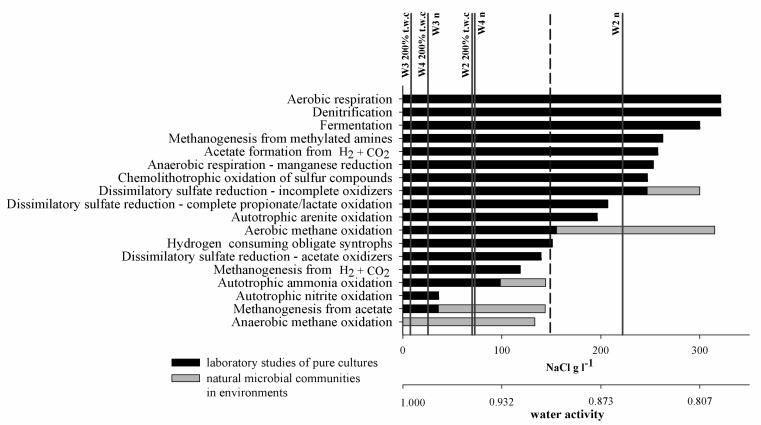
Approximate upper salt concentration limits for the occurrence of selected microbial processes. Dashed line represents 2.5 M NaCl cut-off for extreme halophiles (adapted from Oren [51,52]). Vertical lines represent NaCl concentration in natural moisture samples (W2_n_–W4_n_) and in 200% t.w.c. samples (W2_200%t.w.c_–W4_200%t.w.c_) (current study).

The presence of dormant microbial communities would largely explain the determined relationship between water content and microbial activity, which occurred in each case of the studied processes. Water content and salinity determine water availability for the microbiota, commonly expressed as water activity (aw), which decreases when the concentration of solutes grows. The minimum water activity necessary for cell growth and function is different for different groups of microorganisms. For most microorganisms it is ∼0.9 aw [46,47] but for extremophilic species, the current known limit for life has been observed as ∼0.61 aw [48,49]. In the case of halophilic *Archaea* and *Bacteria*, growth with a water activity < 0.755 has also been observed [49]. Water availability also determines methane-related processes. The most widespread along the aw gradient is aerobic methane oxidation, which is carried out by a range of microbiota and generally increases with increasing water activity, reaching a maximum rate at 0.95 aw [50]. Anaerobic oxidation of methane has been much less examined and confirmed cases are from aquatic habitats with the lowest aw of ~0.932 (Figure 8).

Methane formation is also almost exclusively related to aquatic or water-saturated conditions. This is mostly due to the necessity to protect the main enzymes of methanogenic pathways against oxidative damage. The lowest aw at which methanogenic activity has been detected was in NaCl-rich brine with a water activity as low as 0.741 [53].

However, not all methane formation pathways are equally resistant to salinity. Environmental pressure forces the cells into additional metabolic expenditure. In the case of salinity, this process is associated with the active transportation of ions through membranes or the synthesis of osmolytes. Methanogenic pathways generate very low energy yields. In fact, these microbiota subsist on the edge of survival. In methanogenesis from acetate, ΔG° is −31.1 kJ, from H_2_ and CO_2_ −135.9 or −34.0 kJ, while from methylated amines it is −368.3 from methylamine, −286.5 from dimethylamine and as much as −764.5 kJ from trimethylamine [54]. Consequently, only CH_4_ formation from methylated compounds provides sufficient energy to maintain cell proliferation and thus support an active microbial community in halophilic conditions [55,56].

Incubation of sediments in laboratory conditions with water supplementation caused an increase in water activity, thereby creating more convenient conditions for the metabolic activity of both methanogenic and methanotrophic microorganisms. The prevalence of aerobic methanotrophy over other processes remains in agreement with the described ability of methane-oxidizing microbiota to overcome environmental pressures. Furthermore, the highest values of methane oxidation found in W4 confirm that this sediment has been subjected to diagenesis in conditions that enabled the development of a detectable community of aerobic methanotrophs. In addition, stable isotope composition remains in agreement with the specific microbial activity determined for the sediments. Although the isotopic effect was not large, it suggests that methane oxidation (being also predominantly active in the incubated samples) must have influenced δ^13^C methane values, especially in the W3 and W4 sediments. In the studied samples, none of the taxa related to anaerobic methanotrophy were detected (neither methanotrophic *Archaea* nor symbiotic sulphate reducers). Likewise, in incubation experiments, performed with the addition of sulphate, methane utilization was not observed.

Analysis of results concerning aerobic microbial activity revealed that methane utilization (at water-saturating conditions 200% t.w.c) corresponds greatly with the detected abundance of aerobic methanotrophic bacteria (*Methylomicrobium*, *Methylomonas*, *Methylocystis* and uncultured *Methylocystaceae*) (Figure 3).

### 4.2. Microbial Respiration in Saline Sedimentary Pool

Microbial carbon transformation in saline sediment ecosystems is related not only to the production and oxidation of methane, but also to aerobic and anaerobic respiration of microorganisms, which can affect the concentration of CO_2_ in the atmosphere. Microorganisms are able to degrade a wide range of organic compounds and use carbon to build their own biomass. Our results show that a major controlling factor was also aw. Both aerobic and anaerobic respiration were more intense at 200% t.w.c. At natural moisture, although hampered, microbial activity was also detected.

Generally, anaerobic respiration prevailed over aerobic both in natural and under 200% total water capacity. The highest CO_2_ release was observed in the W3 (siltstone and sandstone) samples.

The TOC content is not the main factor conditioning CO_2_ release. Based on the achieved results, it seems that elevated moisture (200% t.w.c.) was the main factor conditioning CO_2_ release in all investigated samples, although the relation was reversed under aerobic and anaerobic conditions. The limitation in anaerobic microbial organic matter break down was also connected with sample salinity. In the investigated samples, concentrations of NaCl above 72.15 g L^−1^ had an inhibitory effect (Figure 2) although the differences in respiration activity between samples at salinity 24.64 and 72.15 g L^−1^ of NaCl were not important.

The sediments with the highest salinity had the lowest CO_2_ release. However, under anaerobic conditions the trend was the opposite and the highest level of respiration was in the samples with the highest salinity. Aerobic microorganisms (among the investigated samples) seem to be more resistant to salt stress (based on the determined identity) but also less effective in mineralisation of organic matter (as shown by the respiration studies). The highest anaerobic to aerobic respiration ratio (above 4.8) was obtained for the W3 sample, while the lowest was for the most saline rock (W2). These values suggests that anaerobic carbon transformation dominated in the studied environments. Taxonomic analysis suggests that this was mainly caused by the activity of anaerobic representatives of the genera *Finegoldia*, *Roseburia*, *Veillonella,* and facultative anaerobes of the genera *Escherichia*, *Paenibacillus*, *Serratia*, and *Staphylococcus*, which were highly abundant in the studied microbial communities (Figure 3B). The number of microorganisms associated with anaerobic respiration ranged from 14% (W4) to over 25% (W2). Although no relationship has been demonstrated between the abundance of this group of microorganisms and respiration activity, unidentified microorganisms, which constituted a large part of the microbial community identified in our samples, might have played a significant role in respiration.

### 4.3. Microbial Diversity

Microbiological processes such as microbial methane turnover (methanogenesis and methanotrophy) as well as carbon dioxide production (respiration) in aerobic and anaerobic conditions depends on the composition of the microbial community. In the Wieliczka Formation evaporitic sediments, a large part of the community was unclassified and belonged to the genus *Bacillus*.

Many studies suggested that deep biosphere ecosystems are a substantial reservoir of novel, yet uncultured, microbes that have significant implications for biogeochemical carbon cycling [56,57,58,59,60]. Magnabosco and others (2018) estimated that the continental subsurface hosts 2 to 6 × 10^29^ microbial cells, and found that community composition was correlated to sample lithology. However, there are still many questions about the role of the subsurface biosphere in the global carbon cycle. For example, it is unknown why concentrations of organic carbon do not correlate with the concentrations of continental subsurface biomass but are correlated with concentrations of the sub-seafloor biomass. Analysis of the Wieliczka Formation sediments may contribute to filling the gaps in knowledge concerning the biogeochemical processes occurring in the continental subsurface and the related microorganisms. To date, no reliable predictor of species richness in the continental subsurface has been identified on a global scale. However, both the presented results and the literature data confirm that *Bacteria* are more numerous than *Archaea* and that the composition of the community depends on the lithology of the analysed samples [56,57]. A few studies dealing with high salinity subsurface samples suggest that ecosystems characterized by high salinity harbour low biological diversity [58,59]. Our results, achieved by high-throughput sequencing of 16S rRNA gene amplicons, are consistent with these studies. As presented in Table 4, in the most saline sample, there was a sharp decrease in the number of detected taxa in comparison to the remaining materials. Besides the overall trends in biodiversity, the dominant phyla found in the Wieliczka Formation sediments (*Firmicutes* (50–92%) in the W2 and W3 samples, as well as *Proteobacteria* and *Actinobacteria* (10–14% and 8–10% respectively) in the W2 and W4 samples have also been previously noticed in other deep saline ecosystems [59,60].

#### 4.3.1. Prevalence of Unclassified Bacteria in Saline Sedimentary Pool

Apart from the identified taxa, analysis of 16S rRNA amplicons showed a high proportion of unclassified sequences (at the domain level: 26%, 3% and 64%, respectively in the W2, W3 and W4 samples). Analysis of the evolutionary relationships of the most common unclassified sequences using NCBI database data (Figure 5) revealed that they most likely belong to *Bacteria*. Such a significant share of unclassified microorganisms results from the fact that the studied environment is extreme, very poorly understood and could be called a ‘rare biosphere’ [61,62]. These results are not surprising, as 16S rRNA gene sequence libraries, constructed from DNA extracts of environmental samples, often contain a high proportion of unclassified sequences. An example are not only deep subsurface samples such as petroleum reservoirs [63], subsurface fluids [57] and brines [59], but also more available, yet scarcely known, biotopes such as sea sediments [64], floodplain meadows [65] or even heritage objects and buildings [66]. The unclassified sequences evidence the presence of a potentially unique microbial community ecology and the presence organisms with novel taxonomy, so called taxonomic “blind spots” [67].

Comparison of the Wieliczka Formation sediments’ unclassified sequences with the sequences available in the NCBI database showed that the same (100% identity, 100% coverage) or very similar (identity above 95%, 100% coverage) sequences have been found all over the world in many studies. Their occurrence concerns similar, commonly extreme environments, e.g., marine water (Unclassified_WGW232); lava (Unclassified_WGW034, Unclassified_WGW077); groundwater (Unclassified_WGW186); Baltic Sea sediments, open-cast mine, pyrite mine (Unclassified_WGW002) or cave biofilms (Unclassified_WGW149). Some of the analysed sequences occurred in a variety of environments, including different types of soils which are also often contaminated, ANNAMOX bioreactors, waste water, fermented grains and rhizosphere (Table S1). The multitude and variety of environments in which the presence of microorganisms closely related to the Wieliczka Formation inhabitants was confirmed and suggests a continuum between surface and subsurface ecosystems. Geological conditions indicate that it is unlikely that microorganisms in the studied formation could nowadays migrate from surface ecosystems. This continuum has a temporal rather than spatial dimension and relates to the preservation of resting forms of microorganisms in the salty sediments, which were present in the studied sediments at the earlier stages of their formation. Variable origins of the microbiota may explain their versatile metabolic capabilities (revealed by aerobic and anaerobic respiration and potential for methane oxidation and production) as revealed by the microcosm studies (Figure 2).

#### 4.3.2. Ubiquity of *Bacillus*-like Bacteria

Among the most abundant sequences found in the Wieliczka Formation sediments were those assigned to *Bacillus* (from 4.5% in W4 to as much as 91% in W3) (Figure 3). The genus *Bacillus* represents ubiquitous bacteria widely distributed amongst surface environments. They have been isolated from diverse environments such as freshwater, saline waters, soils, plants, animals and air [68,69,70,71]. These bacteria are also known from their adaptive skills and for being isolated from extreme environments [72] including those highly saline [73]. Resistance of *Bacillus* spores to extreme conditions is the result of several strategies. First is the structure of their spores, which are resistant to heat, desiccation and other damaging agents. The second strategy used by *Bacillus* is repair systems. *Bacillus* species produce a large number of extracellular enzymes and their spores are equipped with enzymes of multiple DNA repair pathways [74]. The presence of *Bacillus* in the Wieliczka Formation sediments should, therefore, not come as a surprise. The *Firmicutes* phylum, and in particular the *Bacillus* genus from marine ecosystems, also deserve special attention due to their ecological diversity as well as their physiological, biochemical and molecular properties, which proves their high biotechnological potential, e.g., production of biosurfactants [73,75].

Detailed analysis of *Bacillus*-affiliated sequences from the Wieliczka Formation sediments revealed that they created six clusters (*Bacillus*_CL1-6) (Figure 6). These sequences consistently matched NCBI database environmental sequences from seawater, marine sediment, lake sediment, salt, caves, mines and brines (100% identity, 100% coverage) (Table S2). The similarity of the obtained sequences to the sequences in the database (100% identity, 100% coverage) often indicated that several *Bacillus* species could be assigned to one *Bacillus* cluster (Table S2).

*Bacillus* spp. are aerobic or facultatively anaerobic. The maximum concentrations of NaCl reported to support the growth of *Bacillus* spp. to date is 20% salinity [76]. Among the *Bacillus* clusters extracted from the NGS data from the Wieliczka Formation sediments, it was found that most of them show a similarity to facultative anaerobe species such as *Bacillus licheniformis* (*Bacillus*_CL2); *Bacillus haikouensis* (*Bacillus*_CL3); *Bacillus paramycoides*, *Bacillus toyonensis*, *Bacillus cereus* (*Bacillus*_CL4); *Bacillus altitudinis* (*Bacillus*_CL5) and *Bacillus drentensis* (*Bacillus*_CL6) (Figure 6, Table S2). The high level of CO_2_ production in the Wieliczka Formation samples under water-saturated anaerobic conditions, on average, was higher than in aerobic conditions by more than 36% (Figure 2) and may be related to the high proportion of facultative anaerobe *Bacillus*-like bacteria, which have the ability to live in anaerobiosis.

## 5. Conclusions

In summary, it seems that the Wieliczka Formation sediments are inhabited by a microbial community of versatile metabolic potential, but present-day microbial activity (in natural moisture) is largely hampered, although not completely stopped. Our results deepen the knowledge of the subsurface environment and confirm that *Bacteria* are more numerous than *Archaea* and that the composition of the community may depend on the lithology of the analysed samples. A large part of the microbial community constituted unclassified microorganisms or *Bacillus*-like bacteria, and comparison of their sequences revealed a relation to bacteria inhabiting diverse and extreme environments. Microbial activity (methanogenesis, methanotrophy and respiration) in aerobic and anaerobic conditions in the Wieliczka Formation sediments were highly stimulated by moisture. Our results indicate that if appropriate water conditions occur, anaerobic microbial processes leading to the release of CO_2_ into the atmosphere are more efficient than aerobic ones. Furthermore, the ability to oxidize CH_4_ in the investigated sediments was stimulated by aerobic conditions, and the level of aerobic utilization of methane was significantly higher than the efficiency of anaerobic CH_4_ production. Microbial activity was not correlated with organic carbon content and microbial diversity but was significantly limited by high salinity. Our results revealed that water activity is an important factor regulating the microbial activity of continental subsurface sediments Knowledge on the biodiversity and the processes taking place in saline sediments related to carbon transformation via CH_4_ and CO_2_ can supplement the data on the continental deep biosphere, which is still little understood.

## Figures and Tables

**Table 1:** List of samples, depths and lithological types.

Sample	Lithology	Location in Formation	Depth (m)
W2	Clayey salt (zuber) with veins of fibrous salt and lenses of gypsum and anhydrite	layers in spiza salts	110
W3	Siltstone and sandstone	layers in spiza salts	110
W4	Siltstone with veins of fibrous salt and lenses of anhydrite	layers in green salt	130

**Table 2:** Experimental treatments.

Treatment	Headspace Composition	Solution Composition *
Methane production	N_2_	sterilized ddH_2_O
Anaerobic methane oxidation	10% CH_4_ (*v*/*v*) in N_2_	NaSO_4_ (5mM) in sterilized ddH_2_O
Anaerobic respiration	N_2_	sterilized ddH_2_O
Aerobic methane oxidation	10% CH_4_ (*v*/*v*) in air	sterilized ddH_2_O
Aerobic respiration	ambient air	sterilized ddH_2_O

*—with the exception of the treatment aimed at determining anaerobic methane oxidation, parallel incubations were carried out with rock at natural moisture (no water added) and at 200% t.w.c.

**Table 3:** Physico-chemical properties of the Wieliczka Formation sediments (adapted from Stępniewska et al. [19]).

Parameter		W2		W3		W4	
		Mean	±SD	Mean	±SD	Mean	±SD
EC	mS cm^−1^	114.27	3.58	12.74	0.03	37.30	1.30
TOC	%	3.35	0.04	1.06	0.49	0.76	0.05
N-NO_3_	mg kg^−1^	0.00	0.00	0.00	0.00	0.40	0.02
N-NH_4_	mg kg^−1^	40.70	0.12	37.75	0.15	69.62	0.70
P-PO_4_	mg kg^−1^	0.42	0.10	0.19	0.01	0.18	0.02
S-SO_4_	mg g^−1^	25.84	2.90	18.77	1.09	21.41	2.22
Fe	g kg^−1^	10.21	1.02	106.05	36.89	103.27	52.36
Mn	g kg^−1^	0.43	0.04	2.49	0.10	0.83	0.11
Surface area	m² g^−1^	14.75	0.02	19.96	0.03	20.31	0.06
Pore volume	cm³ g^−1^	0.03	0.01	0.05	0.01	0.05	0.01
Moisture	%	4.32	0.02	10.01	0.11	7.39	0.13
Total water capacity	%	31.52	1.22	51.94	3.34	42.03	1.36
NaCl	g L^−1^	221.02	3.5	24.64	0.00	72.15	1.3

**Table 4:** Sequence alpha diversity and richness estimators of microbial communities.

Sample	Observed	Shannon	Simpson	Simpson D’ (Diversity)	InvSimpson	Fisher
W2	77	3.234	0.872	0.128	7.819	8.246
W3	199	0.621	0.166	0.834	1.199	23.639
W4	242	4.987	0.987	0.013	75.955	29.951

## Data Availability

NGS sequence data have been deposited in the NCBI Sequence Read Archive (SRA) database under BioProject’s ID: PRJNA596092 (https://www.ncbi.nlm.nih.gov/search/all/?term=PRJNA596092, accessed on 9 March 2021).

## Supplementary Materials

The following are available online at https://www.mdpi.com/article/10.3390/biology10080792/s1, Figure S1: Concentration of the CO_2_ and CH_4_ in marine sediments under different moisture (natural–n and saturated–200% t.w.c.) and aeration condi-tions at 20 °C (error bars represent standard deviation, n = 3); Table S1: Unclassified sequences matched with NCBI database sequences, Table S2: *Bacillus* sequences matched with NCBI database sequences. All elected items from NCBI (100% identity, 100% coverage).
