# Supplementary material for: Microbial Involvement in Carbon Transformation via CH4 and CO2 in Saline Sedimentary Pool
Source: Biology (Basel). 2021 Aug 17;10(8):792. doi: 10.3390/biology10080792 (PMC8389658; doi:10.3390/biology10080792)
Supplement: Supplementary file 1 [file biology-10-00792-s001.zip › biology-1303819-supplementary.pdf]

Supplementary Materials

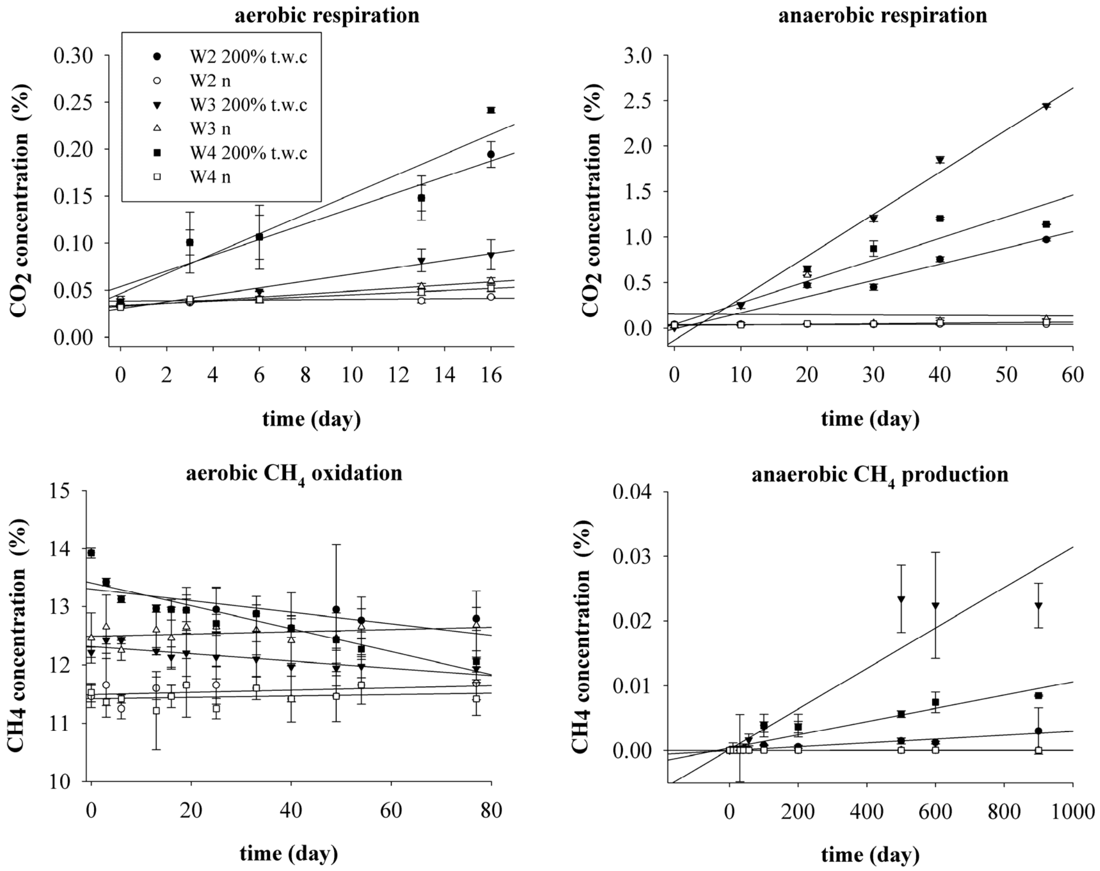

Figure S1. Concentration of the CO_2 and CH_4 in marine sediments under different moisture (natural – n and saturated – 200% t.w.c.) and aeration condi-tions at 20°C (error bars represent standard deviation, n=3).

Table S1. Unclassified sequences matched with NCBI database sequences.

ID	NCBI: Description	Accession number	Identity (%)	source
**Unclassified_ WGW032**	Uncultured bacterium	LN532098.1	100	Denmark: rapid sand filter material of groundwater treatment for drinking water production
	Uncultured anaerobic ammonium-oxidizing bacterium	HQ906638.1	100	China: biofilm reactor
	Uncultured anaerobic ammonium-oxidizing bacterium	AB164467.1	100	Japan: anaerobic biological filtrated reactor
**Unclassified_ WGW025**	Uncultured bacterium	AB775711.1	99.75	Japan: biofilm reactor
	Uncultured bacterium	HQ640583.1	99.75	China: ANNAMOX bioreactor
	uncultured anaerobic bacterium	KP091461.1	98.26	waste water (Chiny)
**Unclassified_ WGW185**	Uncultured bacterium	MG858442.1	100	Fermented grains (China)
	Uncultured bacterium	KF830585.1	100	waste water
	Uncultured organism	HQ791754.1	100	human digestive tract
**Unclassified_ WGW232**	Uncultured bacterium	MH531073.1	100	forest soil (New Zealand)
	Uncultured bacterium	MF968510.1	100	paddy soil (China)
	uncultured bacterium	LT720609.1	100	marine water (Brazil, Atlantic Ocean)
**Unclassified_ WGW034**	uncultured bacterium	MG717054.1	100	Mountain forest soil
	uncultured bacterium	JN616142.1	100	butterscotch organic ooze from lava
	uncultured bacterium	LN715677.1	99.76	Fen soil
**Unclassified_ WGW186**	Uncultured bacterium	HQ640637.1	99.5	ANNAMOX bioreactor
	Uncultured bacterium	KC605909.1	85.43	groundwater
	Uncultured bacterium	JQ376311.2	85.01	soil
**Unclassified_ WGW035**	Uncultured bacterium	KF494600.1	99.50	permafrost soil
	Uncultured bacterium	MN194919.1	98.51	rhizosphere
	Uncultured bacterium	KT790300.1	100	Chiny: rhizosphere
**Unclassified_ WGW002**	Uncultured bacterium	MH524929.1	99.06	forest soil (New Zealand)
	Uncultured bacterium	KC620946.1	98.83	pyrite mine (China)
	uncultured *Gallionella* sp.	KU587047.1	98.59	Eastern Siberia open-cast mine, acidic mine drainage (Russia)
	Uncultured bacterium	KJ616247.1	98.35	Baltic sea Fe-Mn concretions and sediment
**Unclassified_ WGW001**	Uncultured bacterium	LN715646.1	99	Methane-emitting Mire Soils
	Uncultured *Acidobacteria*	HQ598923.1	99	woodland soil
	Uncultured bacterium	JF301231.1	99	boreal forest soil
**Unclassified_ WGW010**	Uncultured bacterium	MH989759.1	100	paddy soil
	uncultured *Methylocystaceae* bacterium	EF072118.1	100	agriculture soil (USA)
	Uncultured bacterium	FJ615931.1	100	agricultural field (USA)
**Unclassified_**	*Streptomyces* sp.	MN093655.1	97.04	pine forest soil

**WGW149**	Uncultured bacterium	KC554093.1	97.04	soil of Yanshan Mountain
	Pseudonocardiaceae bacterium	MH669549.1	96.81	cave silver biofilms (Sanford Underground Research Facility)
**Unclassified_ WGW077**	Uncultured bacterium	HQ899249.1	99.53	Eyjafjallajokull lava
	Uncultured bacterium	FJ532329.1	99.53	PCB-contaminated soil
	Uncultured *Methylophilaceae* bacterium	HE648207.1	99.29	Elodea nuttallii rhyzospheric mercury contaminated sediments

Table S2. *Bacillus* sequences matched with NCBI database sequences. All elected items from NCBI (100% identity, 100% coverage).

ID	NCBI: Description	Accession number	source
***Bacillus*_CL1**	*Bacillus megaterium*	MN133858.1	soil
	Bacillus flexus	MT299641.1	sea water, shellfish, and sediment from the north coast of Java (Indonesia)
	Bacillus flexus	MT279688.1	Benthic Soil, East Kolkata Wetland
***Bacillus*_CL2**	*Bacillus licheniformis*	MN128072.1	Sangameshwar, Tural hotspring (India)
	Geobacillus stearothermophilus	MH729364.1	Compost (India)
	Bacillus sp.	MK120089.1	Salt (Turkey)
***Bacillus*_CL3**	*Bacillus marisflavi*	MT510150.1	Sea sediment (South Korea)
	Bacillus haikouensis	MT299665.1	sea water, shellfish, and sediment from the north coast of Java (Indonesia)
	Bacillus sp.	MT437013.1	Baiyangdian Lake sediment (China)
***Bacillus*_CL4**	*Bacillus paramycoides*	MN134492.1	Caves (India)
	Bacillus toyonensis	MN134491.1	
	Bacillus sp.	MN128021.1	Coalmine soil (India)
	Bacillus anthracis	MN122435.1	Saline source (India)
	Bacillus cereus	LC487876.1	Seawater (India)
***Bacillus*_CL5**	*Bacillus* sp.	MK421402.1	Lithium brines (Chile)
	Bacillus altitudinis	MT373595.1	Stalactite (caves in Hungary)
	Bacillus stratosphericus	MW131050.1	surface of the Mars Science Laboratory
	Bacillus aerius	MW199095.1	Wetland sediments (India)
***Bacillus*_CL6**	*Bacillus mesonae*	MN511751.1	Bamboo Rhizosphere
	Bacillus drentensis	HQ857753.1	soil
	Bacterium HN1-10-48	JN118011.1	flooded paddy soil
